# Feasibility, Acceptability, and Clinical Significance of a Dyadic, Web-Based, Psychosocial and Physical Activity Self-Management Program (*TEMPO*) Tailored to the Needs of Men with Prostate Cancer and Their Caregivers: A Multi-Center Randomized Pilot Trial

**DOI:** 10.3390/curroncol29020067

**Published:** 2022-02-01

**Authors:** Sylvie D. Lambert, Lindsay R. Duncan, S. Nicole Culos-Reed, Laura Hallward, Celestia S. Higano, Ekaterina Loban, Anne Katz, Manon De Raad, Janet Ellis, Melissa B. Korman, Carly Sears, Cindy Ibberson, Lauren Walker, Eric Belzile, Paramita Saha-Chaudhuri, Helen McTaggart-Cowan, Stuart Peacock

**Affiliations:** 1 St. Mary’s Research Centre, Montreal, QC H3T 1M5, Canada; ekaterina.loban@mail.mcgill.ca (E.L.); manon.deraad@ssss.gouv.qc.ca (M.D.R.); cindy.ibberson@ssss.gouv.qc.ca (C.I.); eric.belzile@ssss.gouv.qc.ca (E.B.); 2 Ingram School of Nursing, McGill University, Montreal, QC H3A 0G4, Canada; 3 Department of Kinesiology and Physical Education, McGill University, Montreal, QC H3A 0G4, Canada; lindsay.duncan@mcgill.ca (L.R.D.); laura.hallward@mail.mcgill.ca (L.H.); 4 Health and Wellness Lab, Thrive Centre, Faculty of Kinesiology, Department of Oncology, Cumming School of Medicine, University of Calgary, Calgary, AB T2N 1N4, Canada; nculosre@ucalgary.ca; 5 Department of Psychosocial Resources, Tom Baker Cancer Centre, Calgary, AB T2N 4N2, Canada; 6 Vancouver Prostate Centre, Prostate Cancer Supportive Care Program, Department of Urologic Sciences, University of British Columbia, Vancouver, BC V6T 1Z4, Canada; madronaoncology@gmail.com; 7 Department of Family Medicine, McGill University, Montreal, QC H3S 1Z1, Canada; 8 Cancer Care Manitoba, Winnipeg, MB R3E 0V9, Canada; drannekatz@gmail.com; 9 Odette Cancer Centre, Sunnybrook Health Sciences Centre, Toronto, ON M4N 3M5, Canada; janet.ellis@sunnybrook.ca; 10 Sunnybrook, Research Institute Department of Evaluative Clinical Sciences, Toronto, ON M5S 1A1, Canada; melissa.korman@sri.utoronto.ca; 11 University of Calgary, Calgary, AB T2N 1N4, Canada; carly.sears@albertahealthservices.ca; 12 Department of Oncology & Psychology, University of Calgary, Calgary, AB T2P 4Z6, Canada; lauren.walker@albertahealthservices.ca; 13 Department of Mathematics and Statistics, University of Vermont, Burlington, VT 05405, USA; paramita.saha-chaudhuri@uvm.edu; 14 Canadian Centre for Applied Research in Cancer Control, Cancer Control Research, BC Cancer, Faculty of Health Sciences, Simon Fraser University, Burnaby, BC V5A 1S6, Canada; hcowan@bccrc.ca (H.M.-C.); speacock@bccrc.ca (S.P.)

**Keywords:** self-management, prostate cancer, caregivers, behavior change, physical activity, e-Health interventions, dyadic interventions, psychosocial needs, dyads, pilot study

## Abstract

Background: Prostate cancer is the most common cancer diagnosis among men. Family caregivers (often female spouses) play a key role in ensuring patients’ needs are met, frequently assuming their role with no formal training, which can contribute to a high burden. The purpose of this study was to pilot *TEMPO*—the first dyadic, Tailored, wEb-based, psychosocial and physical activity self-Management PrOgram for men with prostate cancer and their caregivers. Methods: 49 men with prostate cancer and their caregivers were randomized to *TEMPO* or usual care. Baseline and follow-up questionnaires were completed to assess feasibility, acceptability, and clinical significance. A priori benchmarks for these outcomes were set. Thirteen exit interviews were conducted to further explore acceptability. Results: Feasibility benchmarks were met with the exception for recruitment with on average 6.1 dyads recruited/month (benchmark: 8 dyads/month). Benchmarks of acceptability focused on attrition (<25%) and system usability, which were met. Using the strict criteria for adherence of 100% of the module viewed and participants spending at least 15 min on the module, 45% of participants were adherent. The clinical significance on anxiety and quality of life was supported for caregivers, and mostly supported for the men with prostate cancer. Conclusion: This pilot trial was successful, with minor modifications needed prior to a large trial.

## 1. Introduction

Prostate cancer is the most common cancer among men [1]. Despite increasing survival rates, prostate cancer and its treatment remain a major life stressor [2,3], and confront men with many physical symptoms (e.g., urinary incontinence, bowel dysfunction, and sexual dysfunction) [4,5] and psychosocial challenges (e.g., anxiety, depression, and fatigue) [6,7,8]. These challenges often remain poorly managed, resulting in impaired functioning and lower quality of life than the general population [9].

With prostate cancer care mainly delivered as outpatient treatment, most men rely on their partners or family members (referred to as caregivers) for support in coping with daily cancer challenges [10,11]. Caregivers are (more than ever) tasked with complex illness management roles (e.g., managing side effects) [12], usually with little formal training, potentially leading to inefficient trial and error. Although caregiver support positively impacts patients’ health outcomes [13,14] and reduces demands on the health care system [12], the impact on the caregiver is significant. Caregivers report clinically significant anxiety [15], depression [16], fatigue [17], and deteriorating or lower physical health than the population norm [18]. As the health of caregivers deteriorates and they are less able to provide support, patients’ health is adversely affected [19].

Given the challenges faced by both men with prostate cancer and their caregivers, there is a need to support the patient–caregiver dyad in negotiating cancer challenges and optimizing their health outcomes. In fact, dyadic interventions are found to be more efficacious than patient-only or caregiver-only interventions, because of the shared learning that occurs and the interdependency of patients’ and caregivers’ coping and health outcomes [20]. These interventions, mostly based in principles of psychoeducation and self-management, focus on teaching the skills needed to address the challenges of illness and build confidence for enacting those skills to enhance wellness [21,22]. Learning these self-management skills involves changing behaviors by using self-monitoring, goal setting, information seeking, decision making, and action planning [23]. Most of these interventions are delivered by trained health care professionals, in clinic settings or home-visits, over the course of weeks to months. Despite their efficacy [20,24,25,26], as these interventions are resource- and cost-intensive, they are rarely implemented into real-world settings [27]. Therefore, more cost-effective delivery formats are needed.

Web-based interventions can provide efficacious, cost-effective, and tailored self-management support [28]. FOCUS [29] (psycho-education intervention) and CARES [30] (self-management intervention) are two web-based interventions for cancer patients and caregivers that demonstrate feasibility and acceptability evidence for the use of web-based delivery of dyadic interventions. However, limitations of these interventions include (a) the content is pre-determined and not tailored to users’ needs, (b) key components of self-management (e.g., goal setting) are neglected, and/or (c) the focus is often on mental health and not physical health. To address these limitations, we developed *TEMPO*, the first dyadic, **T**ailored, w**E**b-based, psychosocial and physical activity self-**M**anagement **P**r**O**gram specifically for men with prostate cancer and their caregivers.

### TEMPO

*TEMPO* is an evidence-informed program designed for men with prostate cancer and their caregivers (as a dyad) to learn self-management skills based on their priority needs, set goals together, and build confidence to address and manage their needs. Alongside the psychosocial self-management content (e.g., managing stress, symptoms), *TEMPO* supports the use of physical activity as a self-management strategy to enhance overall physical (and mental) health.

Three theoretical frameworks guided the development of *TEMPO*:

1. The Stress and Coping Framework [31], which assumes those who actively cope with cancer challenges report less anxiety [32].
2. The Framework of Dyadic Coping [33] posits positive patient–caregiver coping leads to enhanced outcomes for both members of the dyad [34].
3. Self-Efficacy Theory [35] whereby strategies to enhance self-efficacy were built into *TEMPO* such as behavioral goals, behavior modeling from others, and verbal persuasion.

See Table 1 for a complete description of *TEMPO* following the TIDieR guideline [36]. Briefly, *TEMPO* is a 10-week, web-based intervention, where men with prostate cancer and their caregivers are guided through five modules: needs assessment, goal setting and action planning based on most pressing needs, coping planning, sources of support and motivational tools, and celebrating successes achieved through *TEMPO*. Each module focuses on different aspects of behavior change and self-management skills. The dyads complete worksheets as they move through the modules to apply the skills learned. Additionally, dyads have access to an extensive health library with information on a breadth of topics relevant to the challenges faced by men with prostate cancer and their caregivers.

The development of *TEMPO* has been an iterative process with continuous input from men with prostate cancer, their caregivers, and experts from the field. The preliminary version of *TEMPO* was reviewed by a man diagnosed with prostate cancer and a caregiver for initial feedback. Following this review, content was streamlined, and the sailing metaphor used throughout was toned down. Then, we undertook an acceptability study early on in the development of the intervention, so that any changes could be made before more extensive testing. [37,38]. In the acceptability study [37], dyads endorsed *TEMPO*’s self-directed and dyadic format. The dyads felt the program responded to their needs, and they reported learning new self-management skills, mainly physical activity (less psychosocial self-management strategies) as a way of responding to a range of physical and emotional concerns.

The next step in evaluating *TEMPO* is a pilot study whereby a priori criteria have been established (see Table 2) to determine *TEMPO*’s feasibility and acceptability. The objectives of this pilot randomized controlled trial (RCT) were to (a) examine its feasibility, which includes rates of recruitment and refusal, questionnaire completion (including questionnaires for future cost-utility analysis), and protocol infringement; (b) examine the acceptability of *TEMPO* as evidenced by attrition, adherence, satisfaction, and perceived usefulness; and (c) estimate the clinical significance on anxiety and quality of life (primary outcomes, Supplementary Material S2), as well as depression, self-management skills, physical activity, self-efficacy, and appraisal (secondary outcomes) [39].

## 2. Materials and Methods

### 2.1. Design

Multi-center, stratified, 1:1 parallel, two-group, pilot RCT (NCT04304196), guided by the CONSORT [40] checklist and its adaptation for pilot trials [41].

### 2.2. Participants

A convenience sample of men with prostate cancer and their caregivers (as a dyad) were recruited between April 2020 and February 2021 from six sites in Montreal, Calgary, Toronto, and Vancouver (Canada). Towards the end of the pilot, men alone were also enrolled, as they expressed a dire need for the intervention (these men did have a caregiver with whom they could use *TEMPO*). Ethics approval was obtained from each site. Men with prostate cancer (local or metastasized) were eligible, if they received treatment (i.e., surgery, chemotherapy, radiation therapy, hormone therapy, and/or brachytherapy) within the past two years or were scheduled to receive treatment before the end of the study, and ideally identified a primary caregiver willing to participate. Caregivers were eligible, if they were identified by the man with prostate cancer as the primary source of support, had not been diagnosed with or treated for cancer in the past year, and agreed to participate in the study. Both members of the dyad needed to have access to the internet and understand English or French.

### 2.3. Recruitment Procedures

Participants were recruited by research assistants (RAs) at each site. At some sites, patients were referred to the RA by clinicians, or self-referred after seeing the study poster or pamphlet. At other sites, RAs called directly patients who had previously consented to be contacted about research. Social media were also used to recruit from local community organizations and support groups; interested individuals called local RAs for more information. If the patient was interested, the RA confirmed eligibility of both the patient and caregiver and those eligible completed the online consent form. Once participants consented, they received a link to complete the online baseline questionnaire.

### 2.4. Randomization and Blinding

Upon completion of the baseline questionnaire, the project coordinator randomized dyads (or solo patients), with an allocation ratio of 1:1 to *TEMPO* or usual care, using a computer-generated randomization schedule with random block sizes of 2 or 4, stratified on anxiety (Hospital Anxiety and Depression Scale-Anxiety subscale) [42]. Using an automated interface that was programmed by the study statistician, only the project coordinator was involved in stratification and randomization to ensure allocation concealment and avoid selection bias. The RAs did not have access to the randomization schedule. Participants were not blinded to group allocation, but were blinded to the study outcomes to reduce potential biases.

### 2.5. TEMPO Intervention Group

Participants randomized to *TEMPO* were emailed the website link (https://tempo.truenth.ca/, accessed on 26 November 2021) with a brief, instructional guide for creating an account and navigating the website. Dyads could call an RA, if they needed assistance with account creation and/or navigating the website. See Table 1 for a complete description of *TEMPO* following TIDieR guideline [36].

### 2.6. Wait-List Control Group

Participants randomized to the control group were on a wait list, and given access to *TEMPO* upon return of their follow-up questionnaire.

All participants continued usual care as per the referring center resources and protocols.

### 2.7. Data Collection

Data collection included eligibility screening checklist at the time of recruitment (T0), a baseline questionnaire (T1), and 3-month follow-up questionnaire (T2). All questionnaires were available online on SimpleSurvey in French and English, to be completed by the patients and caregivers individually.

#### 2.7.1. Baseline Questionnaire (T1)

Hospital Anxiety and Depression Scale (HADS) [42] includes 14 items equally divided across the Anxiety and Depression subscales. Subscale scores range from 0–21, with higher scores indicating higher symptoms of anxiety and depression. The Hospital Anxiety and Depression Scale has established reliability in English (α = 0.68–0.93) [43] and in French (α = 0.79–0.89) [44].

The 12-item Short Form Health Survey (SF-12) [45] measures quality of life, and includes a physical component score (PCS) and a mental component score (MSC). Scores are standardized from 0–100, with higher scores indicating higher quality of life. Internal consistency of the physical component score (α = 0.82) and mental component score (α = 0.75) are adequate [46,47]. SF-12 will also be used in future cost-utility analyses of *TEMPO*.

Perceived Stress Scale (PSS) [48] (10 items) to report the frequency of feelings of stress. The total score was used, with higher scores indicating higher stress. This scale has adequate reliability and validity in English (α = 0.74–0.91) [49] and French (α = 0.73–0.81) [50].

Health Education Impact Questionnaire (heiQ v3.0) [51] consists of 40 items across eight subscales: (a) positive and active engagement in life, (b) skill acquisition, (c) constructive attitude, (d) self-monitoring, (e) health services navigation, (f) social integration, (g) health-directed activity, and (h) distress [51]. In line with *TEMPO,* patients responded to the first six subscales, and caregivers responded to the full questionnaire. Higher scores indicate higher levels on that subscale. Reliability is confirmed across subscales (α = ≥ 0.70), in English [52] and French [51,53].

Health Literacy Questionnaire (HLQ) [54] has 44 items, but dyads completed the two most relevant subscales for *TEMPO*: (a) having sufficient information (4 items) and (b) actively managing my health (5 items). Higher scores indicate higher health literacy. This scale has adequate validity and reliability in both languages (α = 0.76–0.94) [54,55].

International Physical Activity Questionnaire-Short Form (IPAQ-SF) [56], a 7-item measure of frequency and duration of vigorous-/moderate-intensity physical activity, walking, and sitting. Activities must be performed for at least 10 minutes. This scale has been found to be valid and reliable, with an overall Spearman’s ρ of 0.80 [56].

Physical Activity Plan and Intention [57], an 8-item questionnaire that measures participants’ indicators of (a) planned physical activity and (b) intentions. On a 7-point Likert-type scale, participants indicated their agreement with the items and higher scores = higher planned physical activity and intention.

Multidimensional Self-Efficacy for Exercise Scale (MSES) [58] (9 items) to assess self-efficacy for exercise participation, including (a) task (e.g., follow directions to complete exercise), (b) coping (e.g., exercise when you lack energy), and (c) scheduling (e.g., include exercise in your daily routine). Higher scores indicate greater self-efficacy. This scale has excellent reliability (α = 0.83–0.91) and validity [58].

Dyadic Coping Inventory (DCI) [59] (37 items, α = 0.63–0.84) [60] to capture how partners support one another in response to individual and collective stressors [61]. This scale has shown to be reliable and valid for use across 25 languages [61]. The total score was used, with higher scores indicating higher levels of dyadic coping.

Revised Dyadic Adjustment Scale (RDAS) [62], a 14-item questionnaire assessing dyadic consensus, satisfaction, cohesion, and affective expression. Higher scores indicate higher dyadic adjustment. Cronbach’s alpha ranged from 0.89 to 0.95 [63]. The measure was optional.

Use of Healthcare services and change in employment (9 items) questionnaire [64] to assess (a) consultation with health care professionals, (b) hospital admissions, (c) medications purchased, (d) use of community services, (e) medical care costs, (f) change in employment, (g) change in hours worked, and (h) performance at work. This questionnaire was included mainly to assess its feasibility for future cost-utility analyses in a larger trial.

Demographics and care information. All participants answered demographic questions, and questions about the cancer diagnosis and its treatment.

#### 2.7.2. Follow-Up (T2) Questionnaires

The follow-up questionnaires included all the measures from T1, as well as a community-based resources survey to assess usual care and co-interventions, and the System Usability Scale [65] (10 items) to assess participants’ view of *TEMPO*’s usability. The average score on the System Usability Scale is calculated where scores above 68 are considered above average. To complement the System Usability Scale, the T2 questionnaire also included a 32-item *TEMPO* feedback survey (based on the concepts of the Technology Acceptance Model [66,67,68]) to identify the strengths and weaknesses of the program (higher scores = positive feedback, see Supplementary Materials S1).

#### 2.7.3. Dyadic Exit Interviews

If at a minimum one member of the dyad returned their T2 questionnaire, the dyad was invited to a semi-structured, telephone or online, exit interview. The purpose of the interview was to further explore *TEMPO*’s perceived usefulness. An interview guide was developed, but questions were tailored to the participants’ *TEMPO* feedback survey answers. The interviews were conducted by the same experienced RA who did them in the initial acceptability study [37].

#### 2.7.4. Study Logs

Study logs were kept by local RAs and the project coordinator to collect data on: (a) number of individuals approached, (b) number of individuals self-referred, (c) number of eligible and ineligible individuals, (d) number of individuals who decline to participate (with reason), (e) number of participants consented and randomized, (f) number of participants who withdrew (with reason), and (g) number of participants who dropped out (with reason).

#### 2.7.5. User Tracking Information

Adherence was measured from user tracking information from the *TEMPO* website, including: number of logins, time spent on each module, number of times logged into each module, modules completed, and worksheets completed within the modules.

### 2.8. Data Analysis

The quantitative data analysis was completed using SAS University Edition [69], STATA 15 [70], and R version 3.1.2 [71].

#### 2.8.1. Feasibility and Acceptability Data

The a priori benchmarks for feasibility and acceptability are detailed in Table 2. Feasibility and acceptability data included recruitment, refusal, attrition, missing data, and adherence rates as well as usability. As part of missing data, the feasibility of collecting data on resource utilization and costs was particularly examined in preparation of a future larger trial. Any protocol infringement and changes made were also noted. Adherence was calculated based on the number of modules completed by each dyad, where a completed module meant that each page was viewed, and it took more than 15 min to complete. Adherence was categorized as high (all 5 modules completed), moderate (3–4 modules completed), and non-adherent (1–2 modules completed). Usability of *TEMPO* was assessed by calculating the average score on the System Usability Scale [65].

#### 2.8.2. Clinical Significance

Baseline characteristics were described for the men with prostate cancer and the caregivers by study group. For each outcome, the effect size was computed as the study group mean difference, divided by the pooled standard deviation [72]. An effect size of at least 0.2 was considered a clinically significant change, given this is a pilot. Moreover, for each outcome, complete data analysis (including dyads and single patients), was performed using the Generalized Estimating Equations approach (GEE) [73]. This approach accounts for the correlation of the dyad outcome and the effect size was computed as the ‘Beta’ estimate of the intervention group in the linear regression model divided by the pooled standard deviation obtained from the unadjusted analysis [74,75]. Additional analyses were performed for the primary outcomes with complete dyad at three months. Additionally, the minimal clinically important difference was calculated for the primary outcomes, striving for 25% of participants improving by the minimal clinically important difference. The minimal clinically important difference is not available for HADS and SF-12 among cancer patients and caregivers, but we relied on validated minimal clinically important difference from other populations: MCID HADS = 1.5 [76], SF-12 PCS = 3.3, and SF-12 MCS = 3.8 [77].

#### 2.8.3. Analysis of Exit Interviews

Interview transcripts were coded by an RA using the NVivo 12 software [78]. Codes were words or statements pertaining to *TEMPO*’s acceptability. To enhance credibility, codes were discussed at regular team meetings. Codes were compared across transcripts to identify key themes.

## 3. Results

### 3.1. Study Participants

**Figure 1:**
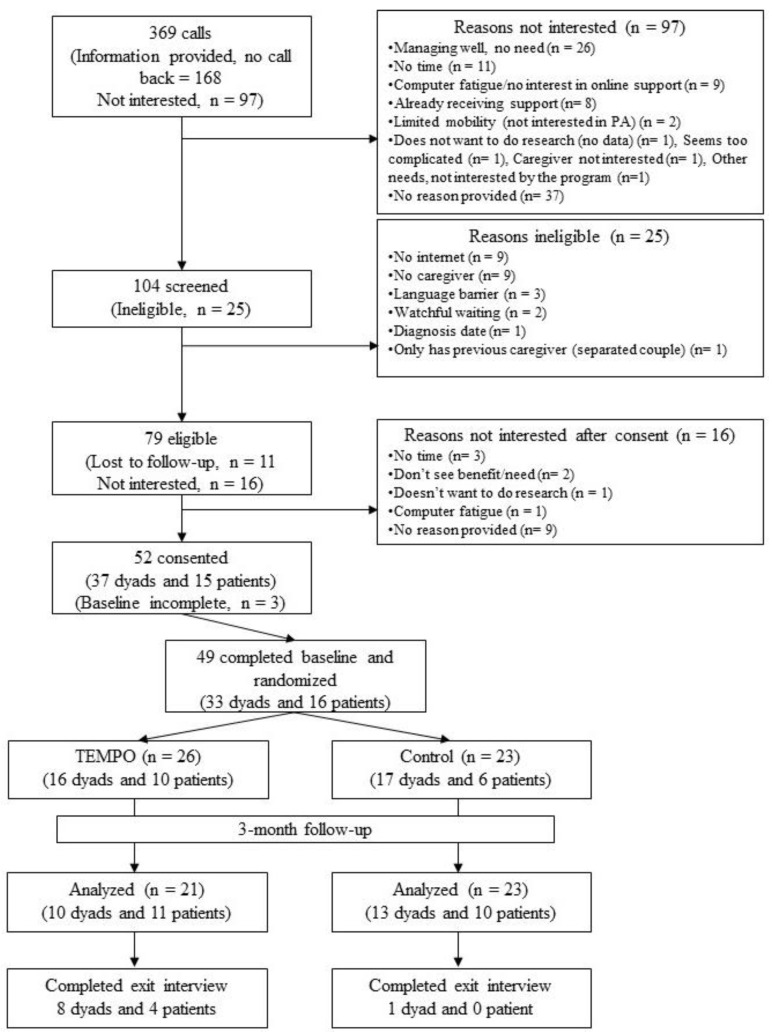
Flow diagram of recruitment and enrollment in *TEMPO*.

Figure 1 details participants’ flow through the study. A total of 33 patient-caregiver dyads and 16 men with prostate cancer were randomized. Table 3 provides a description of the sociodemographic characteristics. Three-quarters of caregivers and men were 61 years old or older and retired. The caregivers were mostly men’s spouses and living with the patient. Two thirds of men were diagnosed with early-stage prostate cancer, and most were at least one year from the diagnosis. The most common co-morbidities for the men and their caregivers were hypertension (29.4–52.6%) and arthritis (18.8–36.8%). Qualitative interviews were conducted, with 12 dyads (in two dyads patients and caregivers were interviewed separately) and one patient who participated without a caregiver.

### 3.2. Feasibility

All feasibility benchmarks were achieved (see Table 2), with the exception of recruitment, with 6.1 dyads (or patients)/month recruited (instead of the benchmark of 8 dyads/month). No major protocol infringements occurred.

#### Feasibility of Cost-Utility Data Collection Methods

The Use of Healthcare Services Questionnaire was found to be feasible and acceptable by participants, suggesting that collecting self-reported resource use and cost data for a subsequent cost-utility analysis will be possible. Participants in the control group reported the following use of health care services—Physician consults: at baseline = 2.50 (SD = 2.42) and at follow-up 3.61 (SD = 3.18) and allied health consults: at baseline = 2.66 (SD = 2.70) and at follow-up 5.94 (SD = 6.66). Participants in the *TEMPO* group reported—Physician consults: at baseline of 1.77 (SD = 1.73) and at follow-up 2.77 (SD = 3.47) and allied health consults: at baseline 2.61 (SD = 3.09) and at follow-up 2.71 (SD = 3.89).

### 3.3. Acceptability

The attrition rate across groups was 19%, below our 25% benchmark. The patients reported a System Usability Scale score of 75 (SD = 15.9), and the caregivers reported a higher score of 81.8 (SD = 15.0). This means that overall, both patients and caregivers reported above average system usability with *TEMPO*. Participants interviewed added that *TEMPO* is user-friendly, straightforward to navigate, and logical in terms of the sequence of modules. One participant explained:

*I liked that the sessions were set up in such a way that it was thought-provoking and engaging. I think sometimes when you go* *through these—like I’ve done 360 processes in business and things like that and you know, your brain starts to drift, but I felt quite engaged throughout the entire session. I thought it was* *very relevant*

(man, 11072–12072)

Suggestions for improvement included: incorporating more options to complete action plans online and enhancing the functionality of certain features for a mobile device.

#### 3.3.1. TEMPO Feedback Survey

Items that scored the lowest on the *TEMPO* feedback survey were (see Supplementary Material S1): The information presented in the health library was new to me, *TEMPO* was applicable to my situation, and *TEMPO* was tailored. During the interviews participants explained that the main reason for the information not being new was that they joined *TEMPO* too late after their diagnosis and they found the information they needed before having access to *TEMPO*. Most participants did not receive the needed information from their health care professionals, rather they undertook independent information-seeking (e.g., internet, library, support groups). This independent learning created doubt about the credibility of the information found, and whether what they were doing was “*correct*”. For these participants, *TEMPO* gave them “*peace of mind*” (man, 1338–1335): *“(TEMPO) reinforced that our approach and attitude were correct and that it had good resources to get further* *information*” (man, 1338–1335). Through using *TEMPO*, a few participants identified some issues that they realized needed to be addressed (e.g., dyadic relationship).

In terms of tailoring, interviewed participants explained that, despite the needs assessment in Module 1 to tailor *TEMPO* content, *TEMPO* was not adapted to their prognosis, time since diagnosis (e.g., library content on treatment decision-making should be hidden if in survivorship phase), fitness level, and the type of support system they had (e.g., Module 4 was not relevant for those with a good support system). One participant explained:

*Different people get on TEMPO at different stages (…) and different people fork in different ways. (…) A person could argue you need 10 different TEMPOs and that just wouldn’t be realistic. But the major branches for me would be first step diagnosis but the person has not yet made a decision about treatment. (…) And then once you’ve made a decision about treatment (…) these are all major branches where people stop having things in common*

(man, 81006–82006)

For caregivers, in particular, learning new skills and using these on a day-to-day basis were items that scored low on the *TEMPO* feedback survey. During the exit interviews, it was clarified that the most frequent skills learned were being more active as a couple and communicating more with each other (especially because of the needs assessment in Module 1). One caregiver said: *We did it together, we would sit down and do the quiz together. I found a couple of things about it by going through it, and realized: “Oh, he hasn’t talked about this, maybe we should talk.” (…) It was beneficial for communication* (caregiver, 32058).

Beyond learning specific self-management skills, what participants found useful were the behavior change modules (more than the factsheets). This is because the modules gave participants a generalizable, clear process or guidelines on addressing needs (often using knowledge they already had) and how to go about integrating PA and other self-management skills. One participant said: *I was engaged by all of the modules. I would say that was the best part of it* *(TEMPO)—because we worked on it together—was the differences in our responses. So, I think, versus any specific* *module I think it was more (…), the process* (man, 11072–12072). Another caregiver said: *TEMPO provides some* *useful guidelines as to how to go about this (working out), and some useful suggestions about different exercise regimens and things to do* (caregiver, 11057–12057).

The *TEMPO* feedback items that scored the highest were (see Supplementary Material S1): I trust the information delivered through *TEMPO*, working through *TEMPO* as a pair made the process more enjoyable (especially for caregivers), and *TEMPO* should be integrated in routine care (especially for the men with prostate cancer). Interviewed participants explained *TEMPO* was the only resource they came across that explicitly involved the caregiver. The greatest value of that was accountability towards the other person of the dyad to achieve goals, and doing things together (e.g., more communication as couples walked together). The different worksheets helped each member of the dyad obtain more insights into how the other was feeling:

*Going through that section (mood-monitoring tool) about how you’re feeling (…) certainly gave me some insight into how (patient) was experiencing* *things. I think we have a very good relationship and we assume we’re on the same wavelength, but that’s not always the case*

(caregiver, 11057–12057)

In a few dyads one partner took the lead and only brought the other member in at critical milestones (e.g., deciding on the action plan) and when they wanted to share relevant information (e.g., one member of the dyad would go through the Health Library and only bring what was relevant to the other member). In other dyads, both partners completed some or all modules sitting in front of a computer together. Most dyads revisited *TEMPO* modules after their initial completion.

#### 3.3.2. Adherence

Three men who were participating alone and two dyads never registered to *TEMPO* (19%). For one dyad, the user tracking did not work. In terms of those who logged in (*n* = 20), 70% (*n* = 14) logged in at least 3 times (mean 5.9 times, SD = 4.9). The modules most often accessed and used for the longest amount time were Module 1 (average log ins 2.8, SD = 2.3; time spent mean = 25.9 min, SD = 24.0 min) and Module 2 (average log ins 2.6, SD = 3.2; time spent mean = 42.3 min, SD = 72.1 min). On average, participants spent 104 min (SD = 129 min) on *TEMPO*. Modules most often completed as defined by 100% pages viewed and spending at least 15 min were also Modules 1 (60%, *n* = 12) and 2 (40%, *n* = 8). Other modules were completed by 10–15% of participants. Using the same criteria for module completion, 1.4/5 modules on average were completed and 45% (*n* = 9) of participants were categorized as adhering to the modules. The worksheet most often completed was the Module 1—unmet needs assessment (*n* = 17, 85%), followed by the Module 2—goal setting worksheet (*n* = 12, 60%). The remaining worksheets were completed by 38% of participants (*n* = 10).

### 3.4. Primary Outcomes

Benchmarks for clinical significance were mostly met. Table 4 presents effects’ sizes for men and caregivers separately and combined. Participants reported clinically significant improvements in anxiety (effect size = 0.24). Anxiety decreased by the minimal clinically important difference in 42.9% of patients and 44.4% of caregivers using *TEMPO*, exceeding rates in the control group (39.1% patients and 30.8% caregivers, Odds Ratio = 1.48).

The clinical significance threshold was also met for quality of life: mental—Effect Size = 0.38 and physical—Effect Size = 0.30. Increases in quality of life-mental equivalent to the minimal clinically important difference was noted for 33.3% of patients, but only 22.2% of caregivers (vs. control patients 26.1% and caregivers 16.7%, Odds Ratio = 1.42). For quality of life-physical, twice as many caregivers in *TEMPO* improved (33.3% caregivers) than in the control group (16.5%). This trend was not observed for patients (*TEMPO* = 23.8%, control 30.4%). In the exit interview, dyads emphasized that many patients were already active, but *TEMPO* engaged more caregivers to be physically active.

A trend is noted in the separate effect sizes for patients and caregivers, whereby caregivers seemed to benefit more from *TEMPO* than patients on mental health outcomes. When removing the men who participated in *TEMPO* alone (i.e., leaving those who completed *TEMPO* as a dyad), effect sizes for the primary outcomes were even higher: anxiety effect size = 0.38, quality of life-mental effect size = 0.48, and quality of life-physical effect size = 0.33 (see Supplementary Material S2). Given the small samples, these results are considered exploratory only. In the exit interviews, dyads referred to coaching each other, to help achieve their goals, which might explain this trend.

### 3.5. Secondary Outcomes

Clinically significant changes were noted for most of the secondary outcomes (see Table 4).

## 4. Discussion

It is now recognized that cancer has a physical and mental impact not only on the individuals diagnosed [2], but also on those who provide them with support—their family caregivers [15,18]. With this recognition, an increasing number of patient–caregiver interventions (or dyadic interventions) have been developed [20,79]. However, within a resource-constrained health care environment (even more so with the pandemic), the intense face-to-face format of these interventions is such that they are rarely translated to routine care. We also know that only 10–15% of patients require high intensity, specialized care to help them cope with cancer challenges [80]. This means that most individuals (and their caregivers) benefit from low-intensity interventions, such as web-based self-management interventions. A Delphi survey [81] found that 86% of managers wanted more research on cost-effective formats for caregiver interventions, including using online formats.

*TEMPO* is the first dyadic Tailored, wEb-based psychosocial and physical activity self-Management PrOgram for men with prostate cancer and their caregivers. As a dyadic intervention, *TEMPO* addresses the needs of men with prostate cancer and their caregivers as well as the relational aspect of coping with cancer challenges [82]. Our initial qualitative acceptability (*n* = 18) study emphasized dyads’ satisfaction with the platform and their perceived benefits of using *TEMPO* (e.g., increased self-management) [37,38]. The present pilot documented key benchmarks for feasibility, acceptability, and clinical significance in preparation for a larger trial. Overall, this pilot was successful, and the four key findings are: (a) dyads favored modules over the factsheets in the health library, (b) a dyadic intervention focused on both psychosocial needs and health behavior was acceptable and might lead to better adherence and outcomes, (c) caregivers seem to benefit more than the men with prostate cancer on mental health outcomes, and (d) more tailoring of *TEMPO* is required.

The first key finding is that dyads found the process-focused behavior change modules more useful than the content-based factsheets. Typical web-based interventions define a series of five or more modules based on pre-determined content, with each module released sequentially [29,30,83]. According to the extensive literature pertaining to patients’ and caregivers’ unmet needs, they generally experience 1–2 dire needs [84,85,86,87]. With typical web-based interventions, this means patients and caregivers potentially have to wait weeks for the release of the most-needed module(s). This in turn might explain some of the high attrition and low adherence observed for web-based interventions. *TEMPO* was designed differently—it is not content-based, but rather focused on the behavior change process inherent in learning new coping and self-management skills, and this seemed particularly useful for participants and appeared to be the reason for achieving the clinical significance benchmarks for most outcomes.

The next key finding was that a dyadic intervention focused on both psychosocial needs and physical activity was acceptable. However, it should be noted that the focus on physical activity was more appreciated than the one on psychosocial needs. This is consistent with Jacobsen et al. [88] who found that a combined stress management and home-based physical activity intervention was more efficacious among patients with cancer than either intervention alone. *TEMPO* is the first dyadic intervention with such a dual focus, challenging the almost exclusive focus on psychosocial needs of current dyadic and caregiver interventions [20]. A systematic review of 14 trials (mostly among caregivers of patient with dementia) concluded that physical activity can increase caregivers’ well-being, quality of life, and self-efficacy [89]. However, many of these studies targeted the caregiver alone, missing an opportunity to involve the patient–caregiver dyad to positively impact adherence and behavior change [90]. Since this review, three dyadic physical activity interventions were published (ballroom dancing [91], dyadic yoga [92], and dyadic strength training [93]), with benefits on patients’ and caregivers’ physical activity levels and quality of life. Only one study targeted men with prostate cancer and their caregivers [93], all used a face-to-face format, and none offered comprehensive content to address psychosocial needs. Of note, the consent rate in the present pilot exceeded those in these studies [91,93], which might support the more flexible approach taken by *TEMPO*.

Adherence to web-based interventions remains a challenge. Low adherence means that individuals might not be exposed to the intervention enough to change outcomes [94]. In the present pilot, only 19% of participants did not complete any modules, much lower than the two-thirds reported in a recent systematic review by Beatty et al. [94]. The literature generally indicates that participants complete on average half of the modules offered [94,95,96,97,98,99,100]. In the present study, 1.7 modules on average were completed, but this is using the stringent criterion that all pages of a module were viewed and at least 15 min was spent on *TEMPO*. Definitions of module completion vary widely across studies, and if we use a less stringent criterion such as 50% of pages viewed and spending at least 5 min on the module, the number of modules completed increases to 2.5. However, focusing simply on number of modules completed assumes that all modules are equally important. We did not define a priori which modules are the most important ones for a therapeutic effect but based on the interviews these would be Modules 1 and 2 where needs assessment and goal setting were the active components. These two modules were completed by 60% and 40%, respectively, of participants. Using less stringent criteria, completion was as high as 70–80%. Men with prostate cancer and the caregivers did report coaching or guiding each other through the intervention, and this form of guidance might increase adherence [94].

The effect sizes mostly met our a priori benchmarks. In examining the individual effect sizes, caregivers seemed to benefit more than the men with prostate cancer on mental health outcomes. Although this is a pilot and such a conclusion needs to be taken with caution, our observation is consistent with other studies finding that caregivers benefit more than patients from a dyadic intervention [101]. This might be because caregivers have access to few supportive care services. Other explanations might be that caregivers felt some relief in their burden by knowing patients are getting support as well (less pressure to provide all the support) or women might feel more comfortable disclosing their anxiety and depression symptoms.

*TEMPO* was designed so that dyads could tailor content based on the needs assessment in Module 1. However, dyads did not perceive this as tailoring, rather interview data indicated that tailoring needed to occur based on stage of disease, time since diagnosis, fitness level, and the type of support systems they had access to. Northouse et al. [29] developed a tailored, web-based psycho-education interventions for patients with cancer and their caregivers, and in this intervention, tailored messaging was developed based on cancer type and dyad relationship as well as level of dyadic communication. A similar strategy could be used in future iterations of *TEMPO* to integrate the suggestions from participants.

### 4.1. Strengths and Limitations

This pilot study tested *TEMPO* using a rigorous design, enrolled dyads across different settings, and demonstrated the acceptability of a process-based (as opposed to content) intervention. Another strength is the clear a priori benchmarks that were established and mostly met. A potential bias relating to participants who declined because they had no time is acknowledged and identifies a marketing strategy that needs to be developed. Another limitation is that the reliance on online, text-based content for the modules might exclude some patient sub-groups (e.g., those with low-literacy levels, those who do not have access to the internet, those who do not speak English or French), and diversifying the modes of delivery (e.g., more videos, images) will be considered in future developments.

### 4.2. Implications

*TEMPO* may be an important tool to support men with prostate cancer and caregivers facing the challenges of prostate cancer that could be integrated in a stepped care approach to supportive care, and eventually adapted to other cancer types. This pilot also emphasized the importance of supporting the behavior change process inherent in self-management (beyond passive dissemination of information). Some improvements will include further tailoring *TEMPO* to the men’s stage of disease, time since diagnosis, fitness level, and the type of support available. In addition, in the larger trial, dyads need to be approached closer to the time of diagnosis.

## 5. Conclusions

This pilot study was successful, and the acceptability, feasibility, and clinical significance of *TEMPO* was supported. The general positive outcomes might be due to several factors. First, the development of *TEMPO* has been an iterative process, with continuous input from men with prostate cancer and their caregivers. Second, *TEMPO* guides dyads through the behavior change process inherent in self-management, setting it apart from many other web-based interventions. Third, the dyadic format might be harnessing the synergistic effect of supporting both patients and caregivers in meeting their needs (which seems to particularly beneficial for caregivers). Even if the results of this pilot are promising, a larger trial needs to be conducted prior to concluding that *TEMPO* is efficacious.

## Figures and Tables

**Table 1:** TIDieR guideline for reporting of interventions.

Items	Description
Brief Name	Tailored, wEb-based, psychosocial and physical activity self-Management PrOgram (*TEMPO*)
Why	*TEMPO* aims to increase dyads’ confidence in using self-management strategies demonstrated to be effective in addressing key psychosocial issues (e.g., dealing with stress) and assist dyads in developing the self-regulatory skills necessary to meet the physical activity guidelines.
What (materials and procedures)	Dyads complete five modules: (a) identification of needs and priorities, (b) setting goals, (c) tracking progress and developing a coping plan, (d) strengthening support systems, and (e) maintaining behavior change beyond *TEMPO*. Modules focus on specific aspects of the behavior change process and integrate key persuasive technology techniques (e.g., primary task support). Each module specifies online (e.g., worksheets to set goals) and offline (e.g., practicing chosen skills) activities. In addition to modules, *TEMPO* includes a health library, incorporating 49 factsheets based on the most up-to-date evidence on self-management and physical activity. The health library includes eight sections: (a) communicating with your health care team, (b) treatment decision-making, (c) dealing with stress and worry, (d) supporting each other, (e) getting the support you need, (f) wanting to feel more fit and healthy, (g) getting on top of symptoms, and (h) caregiving. *TEMPO* is available in French and in English.
Who provided	*TEMPO* is a self-directed intervention, whereby no external guidance is provided. All the support to navigate the intervention is included in its design.
How	Participants randomized to *TEMPO* are sent an email with a brief, illustrated instructional guide on creating a *TEMPO* account, and on accessing the modules. They are also invited to schedule a phone call with a RA to review the registration instructions, receive support with account creation, and/or receive assistance with module access as required. Once dyads use Module 1 to identify their needs, they can use the appropriate factsheets to get ideas for self-management strategies to address these and set their goals accordingly.
Where	Each module becomes immediately available upon completion of the preceding module. If participants are not completing the modules at the expected pace (2 weeks per module), a maximum of two e-mail reminders are sent.
When and How much	As a self-directed intervention, *TEMPO* can be completed where participants prefer, as long as they have an internet connection.
Tailoring	Dyads complete a needs assessment in Module 1 and based on their answers, they can prioritize issues and set goals to work on throughout *TEMPO*.
Modifications	Following our initial acceptability study: (a) new health library content was added to help participants engage in physical activity during COVID-19-related confinement, (b) streamlining the content of the modules, and (c) instead of releasing modules every two weeks, having them available as soon as the previous one is completed.

**Table 2:** A priori feasibility and acceptability benchmarks [39].

Criteria	Benchmarks	Results
**Feasibility**		
Recruitment rate	8 dyads/month across sites	6.1 dyads (or patients)/month
Refusal rate	<45%	34%
Missing data	<10%	<10%
Protocol infringement	Amenable to change	None
**Acceptability**		
Attrition	<25% across groups	19%
Adherence	75% of dyads adhere to modules	45%
System usability (satisfaction)	High system usability reported	Above average
**Clinical Significance**		
Effect size	0.2 on the primary outcomes	Anxiety = 0.24 Quality of life—mental = 0.38 Quality of life—physical = 0.30
Minimal clinically important difference improvement	25% of participants improve on the primary outcomes by at least the minimal clinically important difference	Anxiety = yes Quality of life—mental = yes Quality of life—physical = patient no, caregiver yes

**Table 3:** Patient and caregiver demographics.

Characteristics	Men with Prostate Cancer		Caregivers	
	*TEMPO* (*n* = 26)	Control (*n* = 23)	*TEMPO* (*n* = 16)	Control (*n* = 17)
	*n* (%)	*n* (%)	*n* (%)	*n* (%)
**Age**				
≤60	6 (23.1)	6 (26.1)	4 (25.0)	6 (35.3)
≥61	20 (76.9)	17 (73.9)	12 (75.0)	11 (64.7)
**Sex**				
Male	26 (100)	26 (100)	1 (6.2)	1 (6.2)
Female			15 (93.8)	15 (93.8)
**Language**				
English	23 (88.5)	21(91.3)	15 (93.7)	15 (88.2)
French	0	1 (4.3)	0	0
Other	3 (11.5)	1 (4.3)	1 (6.3)	2 (11.8)
**Country of Birth**				
Canada	20 (76.9)	17 (73.9)	13 (81.3)	12 (70.6)
Other	6 (23.1)	6 (26.1)	3 (18.7)	5 (29.4)
**Education**				
High school or below	0	2 (8.7)	1(6.3)	4 (23.5)
Post-secondary diploma	10 (38.5)	10 (43.5)	4 (25.0)	3 (17.6)
Undergraduate university	6 (23.0)	5 (21.7)	6 (37.5)	5 (29.4)
Graduate diploma	10 (38.5)	6 (26.1)	5 (31.2)	5 (29.4)
**Employment**				
Full time	5 (19.2)	7 (30.4)	1 (12.5)	2 (16.7)
Part time	2 (7.7)	1 (4.3)	5 (62.5)	2 (16.7)
Retired	15 (57.7)	14 (60.9)	2 (25.0)	7 (58.3)
Other	4 (15.4)	1 (4.3)	0 (0.0)	1 (8.3)
(Missing)			(8)	(5)
**Patient-Caregiver Relationship**				
Spouse/Partner	23 (88.5)	20 (87.0)	16 (100)	16 (94.1)
Other (friend, relative, etc.)	3 (11.5)	3 (13.0)	0	1 (5.9)
**Living Together**	22 (84.6)	20 (87.0)	15 (93.8)	16 (94.1)
**Marital Status**				
Married/common law	22 (84.6)	22 (95.7)	15 (93.8)	17 (100.0)
Other	4 (15.4)	1 (4.3)	1 (6.2)	0
**Time Since Diagnosis**				
<6 months	1 (3.8)	1 (4.3)		
6–12 months	5 (19.2)	2 (8.7)		
12–24 months	16 (61.5)	10 (43.5)		
>24 months	4 (15.4)	10 (43.5)		
**Stages**				
Early	16 (61.5)	14 (60.9)		
Advanced	8 (30.8)	9 (39.1)		
Don’t know	2 (7.7)	0 (0.0)		
**Treatment** (can have more than one)				
Surgery	17 (65.4)	16 (69.6)		
Chemo	2 (7.7)	1 (4.3)		
Radio	13 (50.0)	5 (21.7)		
Hormonal	13 (50.0)	9 (39.1)		
Brachytherapy	5 (19.2)	3 (13.0)		
Other	5 (19.2)	3 (13.0)		

**Table 4:** Baseline and post-test scores for primary and secondary outcomes for patients and caregivers.

Outcomes	Men with Prostate Cancer					Caregivers					Combined Effect Size *
	Baseline		Follow-Up		Effect Size	Baseline		Follow-Up		Effect Size	
	*TEMPO* (*n* = 26)	Control (*n* = 23)	*TEMPO* (*n* = 21)	Control (*n* = 23)		*TEMPO* (*n* = 16)	Control (*n* = 17)	*TEMPO* (*n* = 10)	Control (*n* = 13)		
	Mean (SD)	Mean (SD)	Mean (SD)	Mean (SD)		Mean (SD)	Mean (SD)	Mean (SD)	Mean (SD)		
**Primary Outcomes**											
**Quality of Life**											
Mental	48.0 (8.5)	46.1 (9.2)	49.3 (10.3)	46.8 (10.8)	0.23	52.5 (7.1)	50.4 (9.3)	54.7 (5.3)	47.6 (9.6)	0.87	0.38
Physical	50.2 (9.3)	49.3 (12.2)	52.6 (6.1)	49.1 (12.5)	0.35	52.7 (8.0)	51.6 (7.9)	51.1 (9.9)	49.2 (8.6)	0.21	0.30
(missing)	(0)	(0)	(0)	(0)		(0)	(1)	(1)	(0)		
**Anxiety**	5.5 (3.4)	6.3 (4.1)	4.5 (4.2)	5.3 (4.7)	0.19	5.0 (4.1)	4.6 (3.6)	3.4 (2.8)	4.8 (3.3)	0.43	0.24
(missing)	(0)	(0)	(0)	(0)		(0)	(0)	(1)	(0)		
**Secondary Outcomes**											
**Depression**	3.6 (3.4)	5.0 (4.2)	3.7 (4.4)	4.8 (4.3)	0.26	2.3 (2.5)	3.1 (2.2)	1.2 (1.6)	3.4 (3.0)	0.88	0.31
(missing)	(0)	(0)	(0)	(0)		(0)	(0)	(1)	(0)		
**Stress**	13.3 (6.2)	13.4 (6.7)	12.8 (7.1)	12.4 (8.1)	0.05	12.3 (7.7)	14.7 (6.0)	11.0 (5.5)	12.1 (5.3)	0.35	0.04
(missing)	(1)	(1)	(1)	(1)		(1)	(2)	(1)	(1)		
**Self-Management**											
Positive engagement	3.3 (0.5)	3.1 (0.6)	3.4 (0.6)	3.2 (0.6)	0.33	3.4 (0.4)	3.1 (0.4)	3.4 (0.5)	3.1 (0.4)	0.90	0.46
Skill acquisition	3.0 (0.4)	3.1 (0.4)	3.3 (0.4)	3.1 (0.5)	0.44	3.1 (0.5)	2.5 (0.4)	2.9 (0.4)	2.6 (0.4)	0.75	0.48
Constructive attitudes	3.4 (0.5)	3.3 (0.7)	3.4 (0.6)	3.3 (0.7)	0.15	3.3 (0.4)	3.0 (0.4)	3.3 (0.4)	3.3 (0.4)	0.00	0.14
Self-monitoring	3.3 (0.3)	3.2 (0.4)	3.4 (0.4)	3.3 (0.4)	0.25	3.3 (0.4)	2.9 (0.5)	3.2 (0.4)	3 (0.5)	0.43	0.29
Health services navigation	3.3 (0.4)	3.4 (0.5)	3.3 (0.5)	3.4 (0.6)	−0.18	3.2 (0.4)	2.7 (0.4)	3.1 (0.1)	2.6 (0.7)	0.92	0.26
Social integration and support	3.1 (0.5)	3.1 (0.6)	3.2 (0.6)	3.1 (0.6)	0.17	3.1 (0.5)	2.7 (0.6)	3.1 (0.8)	2.6 (0.6)	0.73	0.34
(missing)	(0)	(0)	(1)	(1)		(2)	(7)	(1)	(0)		
**Health Literacy**											
Sufficient information	3.0 (0.5)	3.0 (0.6)	3.1 (0.5)	3.1 (0.6)	0.00	3.2 (0.5)	3.2 (0.5)	3.4 (0.5)	3.1 (0.7)	0.48	0.04
Actively managing	3.1 (0.6)	3.1 (0.4)	3.2 (0.5)	3.1 (0.5)	0.20	3.2 (0.5)	3.1 (0.6)	3.4 (0.6)	3.0 (0.7)	0.61	0.38
(missing)	(1)	(1)	(1)	(1)		(1)	(1)	(1)	(1)		
**Physical Activity** (MET)	1894 (1919)	2365 (3313)	2521 (1471)	2253 (1969)	0.17	1953 (2210)	1410 (1082)	2020 (1812)	1713 (1870)	0.17	0.15
**Physical Activity Plan**	4.2 (2.1)	3.7 (2.2)	4.7 (2.0)	3.5 (1.8)	0.63	4.8 (2.0)	4.3 (1.9)	5.0 (1.4)	4.6 (1.9)	0.24	0.48
(missing)	(1)	(2)	(1)	(1)		(1)	(2)	(1)	(2)		
**Physical Activity Intention**	4.6 (2.0)	3.9 (2.1)	4.8 (1.7)	4.1 (1.5)	0.44	5.2 (1.8)	4.8 (1.9)	4.9 (1.2)	4.4 (1.5)	0.36	0.38
(missing)	(3)	(3)	(1)	(1)		(3)	(4)	(3)	(4)		
**Physical Activity Self-Efficacy**	73.0 (21.5)	66.2 (20.4)	78.5 (16.5)	61.6 (26.2)	0.76	75.7 (20.1)	70.9 (16.6)	81.2 (13.9)	68.1 (21.3)	0.71	0.72
(missing)	(4)	(2)	(1)	(1)		(1)	(2)	(1)	(1)		
**Dyadic Coping**	124.9 (24.7)	128.0 (19.5)	132.8 (14.8)	121.5 (21.5)	0.61	136 (22.1)	121.5 (18.8)	135.2 (19.3)	126.1 (17.1)	0.50	0.63
(missing)	(4)	(2)	(1)	(1)		(1)	(2)	(1)	(2)		
**Dyadic Adjustment**	49.4 (12.2)	47.9 (7.5)	53.2 (6.4)	51.0 (8.2)	0.30	54.5 (9.2)	48.5 (6.7)	54.7 (7.6)	51.3 (5.5)	0.57	0.36
(missing)	(7)	(6)	(9)	(11)		(6)	(6)	(7)	(4)		

Note. Data presented are for the entire sample, including dyads and men with prostate cancer who participated alone. * = Effect Sizes are provided for each man and caregiver, as well as the combined effect size (last column). Clinical significance is based on the combined ES. SD = standard deviation.

## Data Availability

The data presented in this study are available on request from the corresponding author.

## Supplementary Materials

The following are available online at https://www.mdpi.com/article/10.3390/curroncol29020067/s1, Supplementary Material S1: *TEMPO* feedback survey results and Supplementary Material S2: Analysis of primary outcomes only for the dyads.
